# Supplementary figures and images for: The DEAD-box RNA helicase CshA is required for fatty acid homeostasis in Staphylococcus aureus
Source: PLoS Genet. 2020 Jul 30;16(7):e1008779. doi: 10.1371/journal.pgen.1008779 (PMC7392221; doi:10.1371/journal.pgen.1008779)

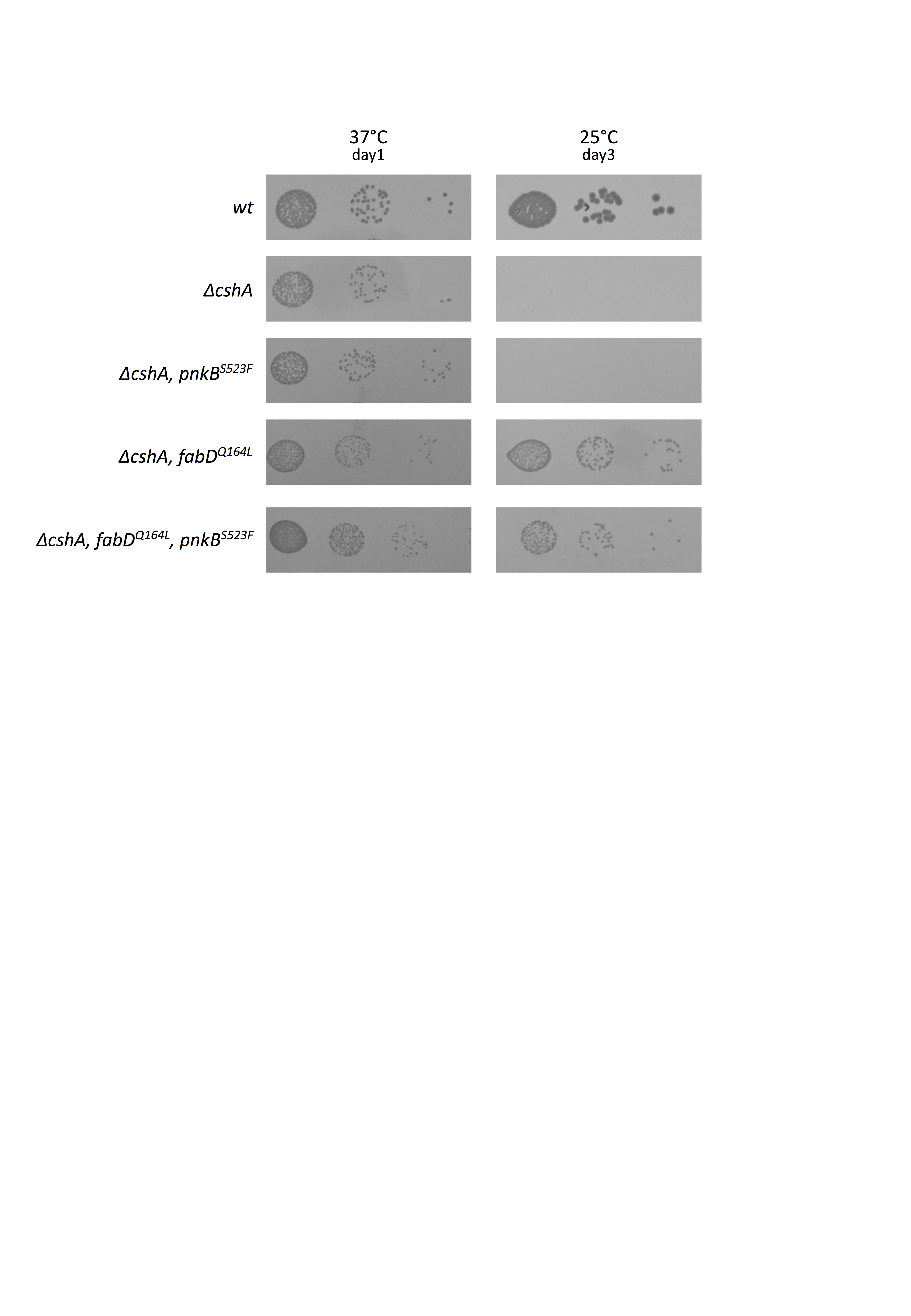

Supplement: S1 Fig — Over-night cultures wt (PR01), ΔcshA (PR01-ΔcshA), ΔcshA/pnkBS523F (SVK87), ΔcshA/fabDQ164L (SVK92) and ΔcshA/fabDQ164L/ pnkBS523F (C53) strains were serially diluted and spotted on MH plates and incubated at 37 C or 25 C. (TIF) [file pgen.1008779.s001.tif]

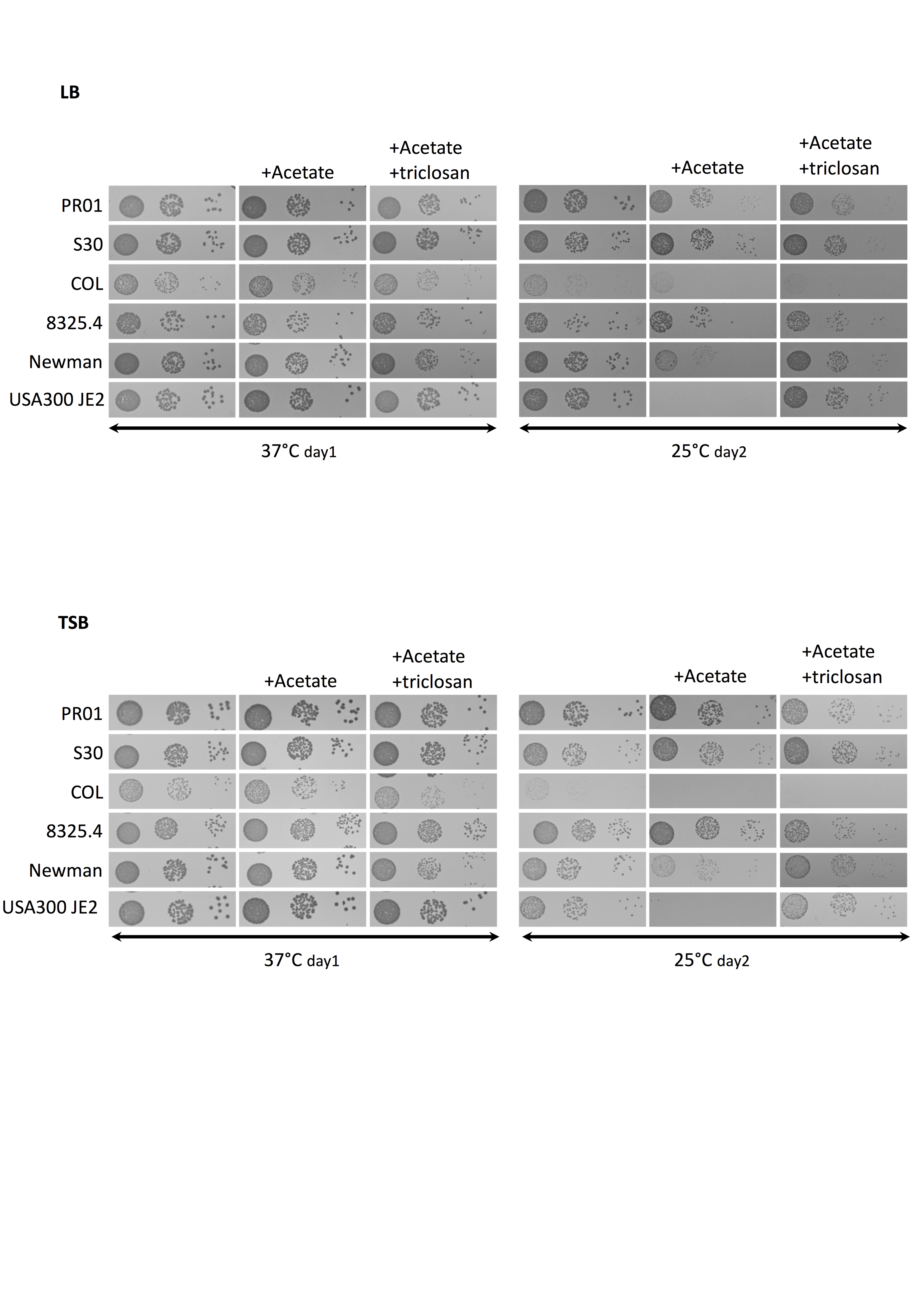

Supplement: S2 Fig — Over-night cultures of various Staphylococcus aureus strains, PR01, S30, COL, 8325.4, Newman and USA300 JE2 were serially diluted and spotted on LB (top panels) or TSB (bottom panels) plates containing when indicated 10 mM Na-acetate and 2 ng/ml of triclosan and incubated at 37°C or 25°C as indicated. (TIF) [file pgen.1008779.s002.tif]

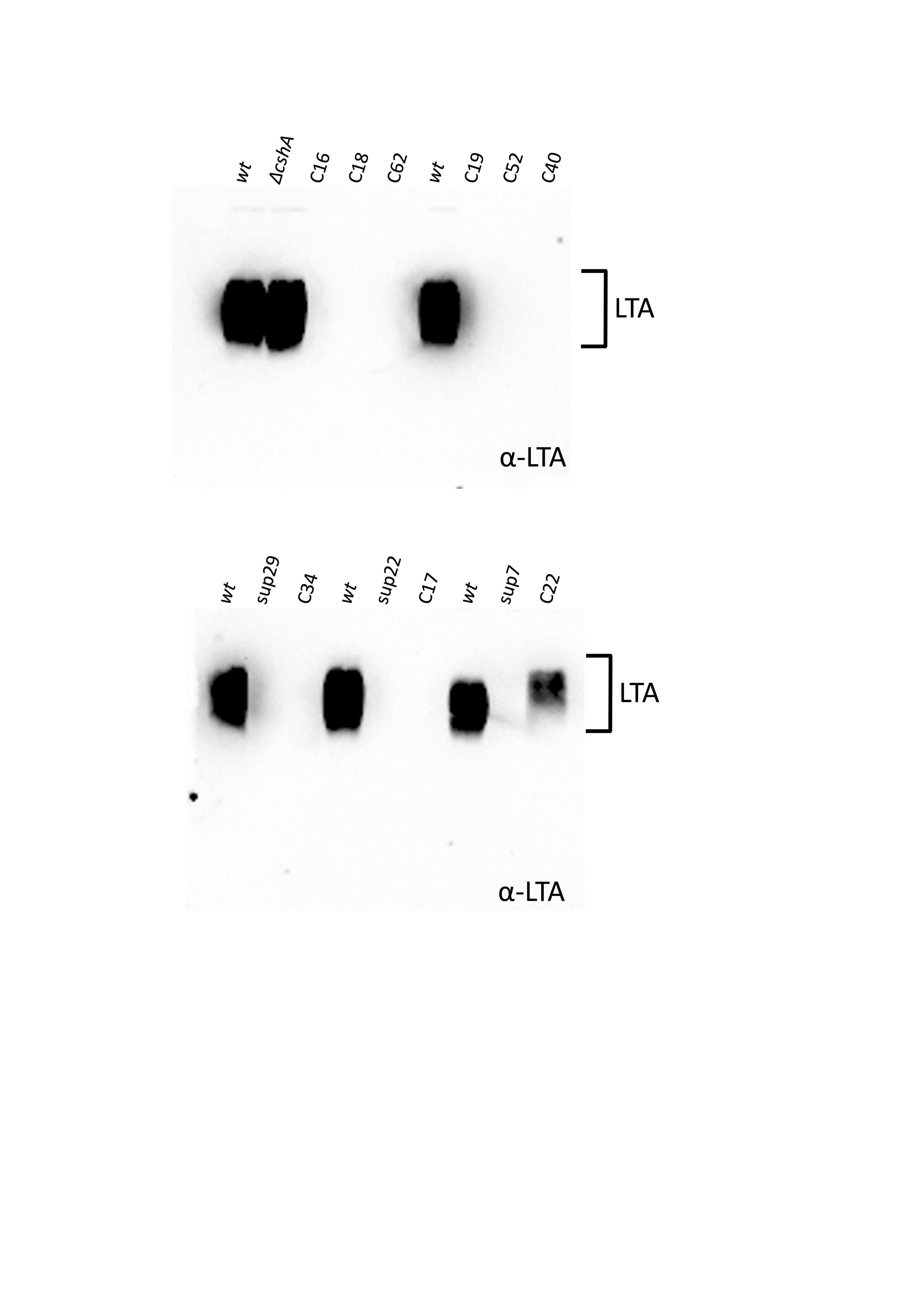

Supplement: S3 Fig — LTA detection by western blot using anti-LTA antibodies in wt (PR01), ΔcshA (PR01-ΔcshA), and the 12 suppressor strains containing mutations in ltaS (sup29, C34, sup22, C17, sup7, C22, C40, C52, C19, C62, C18 and C16). Note that no LTA detection was observed for all ltas mutants except in the C22 strain where LTA production seems to be lower down. (TIF) [file pgen.1008779.s003.tif]

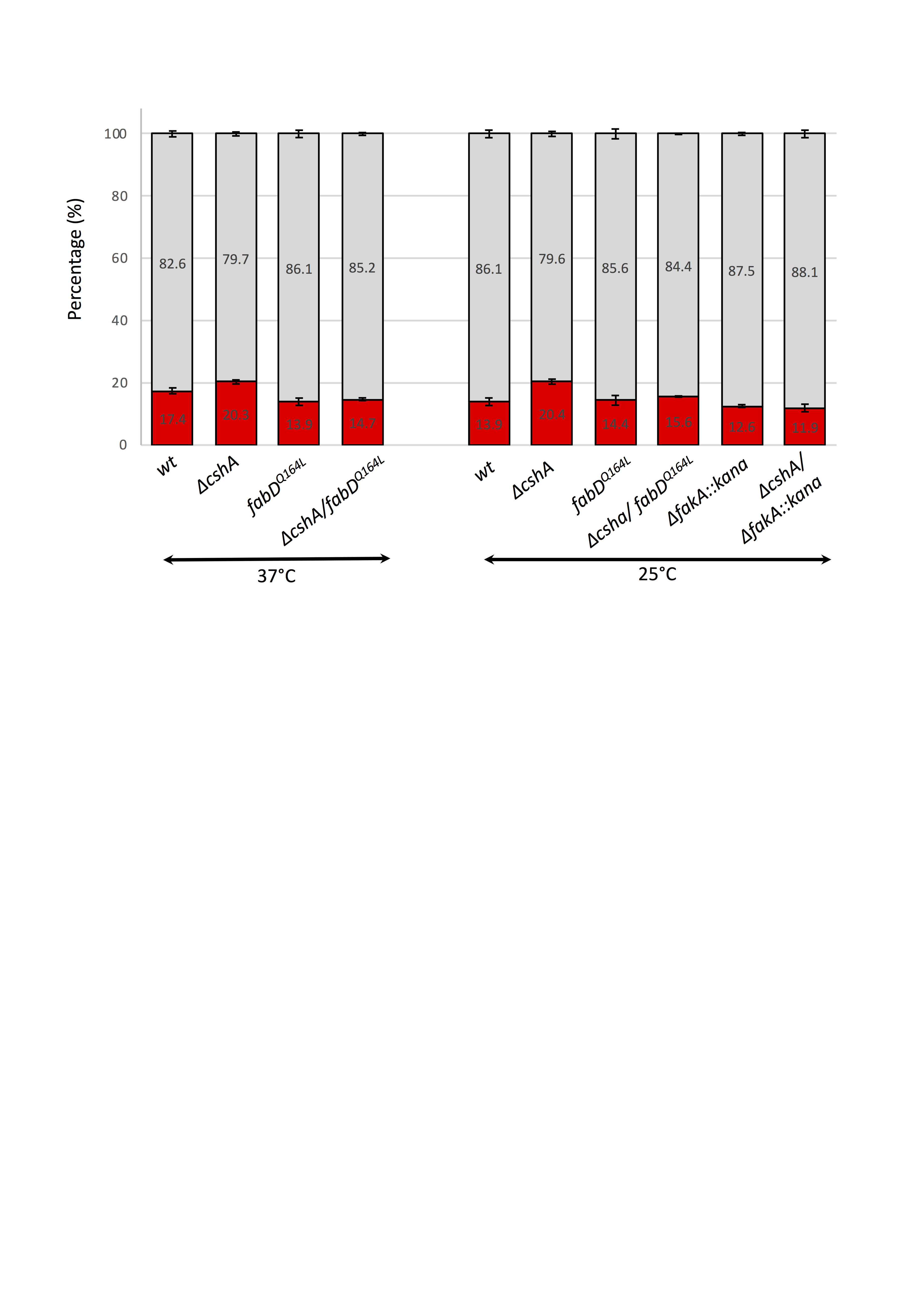

Supplement: S4 Fig — Relative percentage of BCFA and SCFA from wt (PR01), ΔcshA (PR01-ΔcshA), fabDQ164L(SVK86), ΔcshA/fabDQ164L(SVK92 and C53), ΔfakA::kana (SVK47) and ΔcshA/ ΔfakA::kana (SVK48) 37 and 25°C, scrapped on plate. Mean and standards deviation are represented (n = 3 for wt and ΔcshA and 2 for the others, for ΔcshA/fabDQ164L data from SVK92 and C53 were combined). See complete data set in S2 File. (TIF) [file pgen.1008779.s004.tif]
