# Supplementary material for: The DEAD-box RNA helicase CshA is required for fatty acid homeostasis in Staphylococcus aureus
Source: PLoS Genet. 2020 Jul 30;16(7):e1008779. doi: 10.1371/journal.pgen.1008779 (PMC7392221; doi:10.1371/journal.pgen.1008779)
Supplement: S1 Table — (DOCX) [file pgen.1008779.s005.docx]

| **S1 Table: Bacterial strains, plasmids and oligonucleotides.** | | | | | | |
| --- | --- | --- | --- | --- | --- | --- |
| ***S. aureus* PR01 strain and its derivatives ^(1)^** | | | | | | |
| **Name** | **Genotype** | **Description / construction** | | **Source or reference** | | |
| PR01 | *wt* | derivative of clinical strain SA564, *ΔpyrEF* and restriction deficient | | [[1](#_ENREF_1)] | | |
| PR01-ΔcshA | *ΔcshA* | PR01 with *ΔcshA* | | [[2](#_ENREF_2)] | | |
| PR01-*ΔcshA*::kana | *ΔcshA*::kana |  | |  | | |
| PR01-09 | *ΔcshB* | PR01 with *ΔcshB* | | [[1](#_ENREF_1)] | | |
| PR01-03 | *rny^Δ2-24^* | PR01 with *rny^Δ2-24^* | | [[3](#_ENREF_3)] | | |
| SVK7 | *ΔcshA, rny^Δ2-24^* | PR01-ΔcshA *with rny^Δ2-24^* | | [[3](#_ENREF_3)] | | |
| SVK31 | *ΔfapR* | gene replacement of *fapR* using pVK93 in PR01 | | This study | | |
| SVK32 | *ΔfapR, ΔcshA* | gene replacement of *fapR* using pVK93 in PR01-ΔcshA | | This study | | |
| SVK39 | *ΔrpoE* | gene replacement of *rpoE* using pVK104 in PR01 | | This study | | |
| SVK41 | *rpoE^S83_D86 dup^* | gene replacement of *rpoE* using pVK110 in PR01 | | This study | | |
| SVK43 | *ΔltaS::kana* | gene replacement of *ltaS* using pVK107 in PR01 | | This study | | |
| SVK47 | *ΔfakA::kana* | gene replacement of *fakA* using pVK100 in PR01 | | This study | | |
| SVK48 | *ΔfakA::kana, ΔcshA* | gene replacement of *fakA* using pVK100 in PR01 | | This study | | |
| SVK86 | *fabD^Q164L^* | gene replacement of *fapR* using pVK146 in PR01 | | This study | | |
| SVK87 | *pnkB^S523F^* | gene replacement of *pnkB* using pVK150 in PR01-ΔcshA | | This study | | |
| SVK88 | *pdh^A2P^* | gene replacement of *pdhA* using pVK170 in PR01 | | This study | | |
| SVK92 | *fabD^Q164L^, ΔcshA* | gene replacement of *fapR* using pVK146 in PR01-ΔcshA | | This study | | |
| SVK109 | *Δcrt* | gene replacement of *crtOPQMN* using pVK219 in PR01 | | This study | | |
| SVK110 | *Δcrt, ΔcshA* | gene replacement of *crtOPQMN* using pVK219 in PR01-ΔcshA | | This study | | |
| SVK115 | *nfu^G>A -13nt^* | gene replacement of *nfu* using pVK171 in PR01 | | This study | | |
| SVK116 | *lspA^R51H-FsX11^* | gene replacement of *lspA* using pVK173 in PR01 | | This study | | |
| SVK119 | *ltaS^G324stop^* | gene replacement of *ltaS* using pVK231 in PR01 | | This study | | |
| SVK120 | *ltaS^D349Y^* | gene replacement of *ltaS* using pVK232 in PR01 | | This study | | |
| SVK123 | *ltaS^G348D^* | gene replacement of *ltaS* using pVK235 in PR01 | | This study | | |
| SVK124 | *ndhF^V267E^* | gene replacement of *ndhF* using pVK236 in PR01 | | This study | | |
| SVK125 | *ndhF^A223P^* | gene replacement of *ndhF* using pVK237 in PR01 | | This study | | |
| SVK126 | *ndhF^ΔG-8^* | gene replacement of *ndhF* using pVK238 in PR01 | | This study | | |
| SVK127 | *ndhF^S263E^* | gene replacement of *ndhF* using pVK239 in PR01 | | This study | | |
| SVK128 | *ahrC^R44C^* | gene replacement of *ahrC* using pVK227 in PR01 | | This study | | |
| SVK131 | *accD^A164V^* | gene replacement of *accD* using pVK245 in PR01 | | This study | | |
| SVK132 | *accD^F253^* | gene replacement of *accD* using pVK246 in PR01 | | This study | | |
| SVK133 | *accD^A164E^* | gene replacement of *accD* using pVK247 in PR01 | | This study | | |
| SVK134 | *accC^M385V^* | gene replacement of *accC* using pVK249 in PR01 | | This study | | |
| **Suppressors strains selected in the screen ^(2)^** | | | |  | | |
| **name** | ***Genotype*** | | **Pool number** | **source** | | |
| C1 | *accD^A164E^* | | GHU-62 | This study | | |
| C3 | *fakA^P37L^* | | GHU-56 | This study | | |
| C4 | *fakA^K378N-FsX14^* | | GHU-55 | This study | | |
| C5 | *fakA^D38GLY^* | | GHU-55 | This study | | |
| C6 | *fakA^L133H^* | | GHU-56 | This study | | |
| C7 | *nfu^G>A -13nt^* | | GHU-60 | This study | | |
| C8 | *lspA^R51H-FsX11^* | | GHU-62 | This study | | |
| C9 | *ahrC^N31Stop^* | | GHU-62 | This study | | |
| C10 | *fakA^Q483stop^* | | GHU-55 | This study | | |
| C12 | *pdhC^D42Y^* | | GHU-56 | This study | | |
| C13 | non-detected | | GHU-61 | This study | | |
| C14 | *fakA^Ins_IS1181^ ^at +1453pbs^* | | GHU-55 | This study | | |
| C15 | *fakA^T330 _ G341del^* | | GHU-55 | This study | | |
| C16 | *ltaS^K7E_FsX1^* | | GHU-57 | This study | | |
| C17 | *ltaS^R183C^, SA0427^I152F_FsX6^* | | GHU-60 | This study | | |
| C18 | *ltaS^K7E_FsX1^* | | GHU-57 | This study | | |
| C19 | *ltaS^G324Stop^* | | GHU-57 | This study | | |
| C20 | *fakA^G204_ K206 del^* | | GHU-61 | This study | | |
| C21 | *fakA^R454C^* | | GHU-61 | This study | | |
| C22 | *ltaS^T99K/T142K/P156T^* | | GHU-57 | This study | | |
| C23 | *rpoE^S83_D86 dup^* | | GHU-55 | This study | | |
| C24 | *fakA^D268_G299del^* | | GHU-55 | This study | | |
| C26 | *fakA^P365H^, SA0119^N323Y^, SA0901^ΔP301_N306^* | | GHU-62 | This study | | |
| C27 | *fakA^V28stop^* | | GHU-55 | This study | | |
| C28 | *fakA^D29E^* | | GHU-58 | This study | | |
| C29 | non-detected | | GHU-61 | This study | | |
| C30 | non-detected | | GHU-62 | This study | | |
| C31 | non-detected, *SA0679^P288H^* | | GHU-61 | This study | | |
| C32 | non-detected | | GHU-60 | This study | | |
| C33 | *fakA^A467V^, SA1558^I303M^* | | GHU-61 | This study | | |
| C34 | *ltaS^D349Y^* | | GHU-60 | This study | | |
| C35 | *fakA^ins_IS1181^ ^at +1636pbs^* | | GHU-61 | This study | | |
| C36 | *fakA^A18P^* | | GHU-62 | This study | | |
| C37 | *fakA^L133H^* | | GHU-61 | This study | | |
| C38 | *fakA G>A-12nt (in SD)* | | GHU-55 | This study | | |
| C39 | *fakA^ΔR314_H322^, SA1531^S126L^* | | GHU-61 | This study | | |
| C40 | *ltaS^F64L-FsX2^* | | GHU-60 | This study | | |
| C41 | Not detected | | GHU-62 | This study | | |
| C42 | Not detected | | GHU-61 | This study | | |
| C43 | *pdh^A2P^* | | GHU-63 | This study | | |
| C44 | *fakA^G359V^* | | GHU-63 | This study | | |
| C45 | *pdhA^G126D^* | | GHU-63 | This study | | |
| C46 | *fakA^E89K^* | | GHU-63 | This study | | |
| C47 | *fakA^E538Stop^* | | GHU-59 | This study | | |
| C48 | *SA1068^-325_-226del^* | | GHU-63 | This study | | |
| C49 | *fakA^P127L^* | | GHU-63 | This study | | |
| C50 | *fakA^S83T^, SA2299^Q481H^* | | GHU-59 | This study | | |
| C51 | *accC^M385V^, SA2332^S41C^* | | GHU-59 | This study | | |
| C52 | *ltaS^G324Stop^* | | GHU-60 | This study | | |
| C53 | *fabD^Q164L^, pnkB^S523F^* | | GHU-62 | This study | | |
| C55 | *pdhB^P118L^* | | GHU-63 | This study | | |
| C56 | *not detected* | | GHU-63 | This study | | |
| C57 | *ahrC^R44C^* | | GHU-59 | This study | | |
| C58 | *birA^D320F-FsX28^* | | GHU-62 | This study | | |
| C59 | *fakA^E405Stop^* | | GHU-59 | This study | | |
| C60 | *fakA^S366K^, SA1639^T206T^* | | GHU-59 | This study | | |
| C61 | *Not detected* | | GHU-63 | This study | | |
| C62 | *ltaS^K7E_FsX1^* | | GHU-57 | This study | | |
| C63 | *fakA^E361Stop^* | | GHU-59 | This study | | |
| C64 | *fakA^E118 _V183 del^* | | GHU-59 | This study | | |
| C65 | *fakA^R314_H322 del^* | | GHU-63 | This study | | |
| C66 | *bioY^P123R-FsX1^* | | GHU-63 | This study | | |
| sup1 | *birA^R280stop^, SA0099^I242F^* | | GHU-62 | This study | | |
| Sup2 | *fakA^E346­_P365dup^* | | GHU-58 | This study | | |
| Sup3 | *fakA^L133H^* | | GHU-56 | This study | | |
| Sup4 | *fakA^V28stop^* | | GHU-58 | This study | | |
| Sup5 | *fakA^N82K^* | | GHU-58 | This study | | |
| Sup7 | *ltaS^N309Y^* | | GHU-57 | This study | | |
| Sup8 | *fakA^Q483stop^, SA1695^P2P^* | | GHU-58 | This study | | |
| Sup10 | *ndhF^V267E^, SA1815^A311V^, SA0675^G351E^* | | GHU-56 | This study | | |
| Sup12 | *ndhF^A223P^* | | GHU-55 | This study | | |
| Sup13 | *SA1524^insIS1181 at -395pbs^* | | GHU-58 | This study | | |
| sup16 | *accD^A164V^ , SA2135^C>T at -138^* | | GHU-58 | This study | | |
| sup17 | *accD^F253V^* | | GHU-58 | This study | | |
| Sup18 | *SA1068^G35V^* | | GHU-56 | This study | | |
| Sup22 | *ltaS^T352I^* | | GHU-60 | This study | | |
| Sup23 | *ndhF^ins_IS1181 at-12pbs^* | | GHU-56 | This study | | |
| sup24 | *ndhF^ΔG-8^* | | GHU-56 | This study | | |
| sup26 | *pdhA^E362Stop^* | | GHU-56 | This study | | |
| Sup27 | *ndhF^S263F^* | | GHU-62 | This study | | |
| Sup29 | *ltas^G348/D^* | | GHU-56 | This study | | |
| sup30 | *accC^T183I^* | | GHU-62 | This study | | |
| **Derivatives of suppressors strains(3)** | | | | | | |
| **Name** | ***Genotype*** | **Construction** | | | | **references** |
| C51-ΔfapR | *ΔfapR, accC^M385V^* | gene replacement of *fapR* using pVK93 in C51 | | | | This study |
| C1-ΔfapR | *ΔfapR, accD^A164E^* | gene replacement of *fapR* using pVK93 in C1 | | | | This study |
| C43-ΔfapR | *ΔfapR, pdh^A2P^* | gene replacement of *fapR* using pVK93 in C43 | | | | This study |
| **Other *S. aureus* strains** | | | | | | |
| **name** | **Source or reference** | | | | | |
| USA300 JE2 | [[4](#_ENREF_4)] | | | | | |
| COL | [[5](#_ENREF_5)] | | | | | |
| Newman | [[6](#_ENREF_6)] | | | | | |
| S30 | [[7](#_ENREF_7)] | | | | | |
| 8325.4 | [[8](#_ENREF_8)] | | | | | |
| ***E. coli* strain** |  |  |  | | |  |
| **Name** | **Description** | | | | | **Source** |
| DH5α | Standard cloning strain | | | | | lab strain |
| **Plasmids** | | | | | | |
| **name** | **description** | **construction** | | | | **references** |
| **Expressing plasmids** | | | | | | |
| pCN47 | Shuttle vector carrying pT181copwt repC and an  erythromycin resistance cassette | | | | | [[9](#_ENREF_9)] |
| pVK102 | p^HU^-fapR | fusion PCR with fragment1 (OVK221-OVK222 using PR01 as template) and fragment2 (OVK223-OVK184 using PR01 as template) using primer OVK221-OVK184, was cloned in BamHI-AscI in pCN47 | | | | This study |
| pVK174 | p-ahrc^wt^ | PCR fragment with OVK471-OVK472 using PR01 as template was cloned in BamHI-AscI in pCN47 | | | | This study |
| pVK176 | p-ahrc^R44C^ | PCR fragment (OVK471-OVK472) using C57 as template, was cloned in BamHI-AscI in pCN47 | | | | This study |
| pVK197 | p-BCKD | PCR fragment (OVK542-OVK474) using PR01 as template was cloned in BamHI-AscI in pCN47 | | | | This study |
| pVK203 | p-PDH | PCR fragment (OVK544-OVK545) using PR01 as template, was cloned in BamHI-AscI in pCN47 | | | | This study |
| **Plasmids for chromosomal allelic exchange^(3)^** | | | | | | |
| pRLYC8 | empty vector for gene replacement | | | | | [[1](#_ENREF_1)] |
| pVK93 | *ΔfapR* | fusion PCR with fragment1 (OVK176-OVK177 using PR01 as template) and fragment2 (OVK178-OVK179 using PR01 as template) using primer OVK176-OVK179, cloned in BamHI-XhoI in pRLYC8 | | | | This study |
| pVK100 | *ΔfakA::Kana* | fusion PCR with fragment1 (OVK140-OVK146 using PR01 as template), fragment2 (OVK142-OVK143 using PR01,Dcsha::kana as template) and fragment 3 (OVK145 and OVK141 using PR01 as template) using primer OVK140-OVK141, cloned in BamHI-PstI in pRLYC8 | | | | This study |
| pVK104 | *ΔrpoE* | fusion PCR with fragment1 (OVK229-OVK231 using PR01 as template) and fragment2 (OVK232-OVK230 using PR01 as template) using primer OVK229-OVK230, cloned in BamHI-XhoI in pRLYC8 | | | | This study |
| pVK107 | *ΔltaS::kana* | fusion PCR with fragment1 (OVK123-OVK147 using PR01 as template), fragment2 (OVK142-OVK157 using PR01-ΔcshA::kana as template) and fragment 3 (OVK148 and OVK149 using PR01 as template) using primer OVK123-OVK149, cloned in BamHI-PstI in pRLYC8 | | | | This study |
| pVK110 | *rpoE^S83_D86dup^* | PCR fragment (OVK229-OVK230 using C23 as template), cloned in BamHI-XhoI in pRLYC8 | | | | This study |
| pVK146 | *fab^DQ164L^* | PCR fragment (OVK292-OVK293 using C53 as template), cloned in BamHI-XhoI in pRLYC8 | | | | This study |
| pVK150 | *pknB^S523F^* | PCR fragment (OVK290-OVK291 using C53 as template) , cloned in BamHI-XhoI in pRLYC8 | | | | This study |
| pVK170 | *pdh^A2P^* | PCR fragment (OVK475-OVK476 using C43 as template), cloned in BamHI-XhoI in pRLYC8 | | | | This study |
| pVK171 | *nfu^G>A-13^* | PCR fragment (OVK477-OVK478 using C7 as template) , cloned in BamHI-XhoI in pRLYC8 | | | | This study |
| pVK173 | *lspA^R51H-FsX11^* | PCR fragment (OVK481-OVK482 using C8 as template), cloned in BamHI-XhoI in pRLYC8 | | | | This study |
| pVK219 | *Δcrt* | fusion PCR with fragment1 (OVK591-OVK592 using PR01 as template), fragment2 (OVK142-OVK588 using PR01-ΔcshA::kana as template) and fragment 3 (OVK600 and OVK594 using PR01 as template) using primer OVK591-OVK594, cloned in BamHI-XhoI in pRLYC8 | | | | This study |
| pVK227 | *ahrC^R44C^* | PCR fragment (OVK581-OVK583 using C57 as template), cloned in BamHI-XhoI in pRLYC8 | | | | This study |
| pVK231 | *ltaS^G324stop^* | PCR fragment (OVK123-OVK149 using C19 as template), cloned in BamHI-PstI in pRLYC8 | | | | This study |
| pVK232 | *ltaS^D349Y^* | PCR fragment (OVK123-OVK149 using C34 as template), cloned in BamHI-PstI in pRLYC8 | | | | This study |
| pVK235 | *ltaS^G348D^* | PCR fragment (OVK123-OVK149 using sup29 as template), cloned in BamHI-PstI in pRLYC8 | | | | This study |
| pVK236 | *ndhF^V267E^* | PCR fragment (OVK617-OVK618 using sup10 as template), cloned in BamHI-XhoI in pRLYC8 | | | | This study |
| pVK237 | *ndhF^A223P^* | PCR fragment (OVK617-OVK618 using sup12 as template), cloned in BamHI-XhoI in pRLYC8 | | | | This study |
| pVK238 | *ndhF^ΔG-8^* | PCR fragment (OVK617-OVK618 using sup24 as template), cloned in BamHI-XhoI in pRLYC8 | | | | This study |
| pVK239 | *ndhF^S263F^* | PCR fragment (OVK617-OVK618 using sup27 as template), cloned in BamHI-XhoI in pRLYC8 | | | | This study |
| pVK245 | *accD^A164V^* | PCR fragment (OVK621-OVK622 using sup16 as template), cloned in BamHI-XhoI in pRLYC8 | | | | This study |
| pVK246 | *accD^F253V^* | PCR fragment (OVK621-OVK622 using sup17 as template), cloned in BamHI-XhoI in pRLYC8 | | | | This study |
| pVK247 | *accD^A164E^* | PCR fragment (OVK621-OVK622 using C1 as template), cloned in BamHI-XhoI in pRLYC8 | | | | This study |
| pVK249 | *accC^M385V^* | PCR fragment (OVK623-OVK624 using C51 as template), cloned in BamHI-XhoI in pRLYC8 | | | | This study |
| **Oligonucleotides** | | | | | | |
| **For cloning** | | | | | | |
| **name** | **Sequence 5’-3’** | | | | | |
| OVK123 | CTCTCTGGATCCAGAATGCAATTAGAAATGATGAAATAATATTTGGTACGT | | | | | |
| OVK140 | GCTAGGATCCATCTACGTTTAGTAGAGTTTAAAGCGT | | | | | |
| OVK141 | CGTACTGCAGTAATTTCCTGTTTAACTCTATTCCACTTACC | | | | | |
| OVK142 | ATGGCTAAAATGAGAATATCACCGGAATTGA | | | | | |
| OVK143 | TTTTTTATTTTAAATTTTTAAAACAATTCATCCAGTAAAATATAATATTTTATTTTCTCC | | | | | |
| OVK145 | ACTGGATGAATTGTTTTAAAAATTTAAAATAAAAAACTACCAATGATAAATCATCAGTTG | | | | | |
| OVK146 | TCAATTCCGGTGATATTCTCATTTTAGCCATTTCAAGTTGTCCTCCTAAGCTTTCTTGC | | | | | |
| OVK147 | TCAATTCCGGTGATATTCTCATTTTAGCCATGATTCTTTCCCCCGTTATTTAGATAATAAATCTTGC | | | | | |
| OVK148 | TTTACTGGATGAATTGTTTTAATATTTAAACACGAACTCGGATTGATAAAATATCAATCC | | | | | |
| OVK149 | CGTACTGCAGCATCTATCATTCGCTTGAACAATTTATCAC | | | | | |
| OVK157 | AGTTCGTGTTTAAATATTAAAACAATTCATCCAGTAAAATATAATATTTTATTTTCTCCC | | | | | |
| OVK176 | ATACGGATCCGGTGAGATGGATGTGTCTTCAATTAAAC | | | | | |
| OVK177 | ACGTTTATCTTTCTTTAGTTTCAACGTCT | | | | | |
| OVK178 | AGACGTTGAAACTAAAGAAAGATAAACGTAAAATGTTTTATGATAAGCGAGGATAAAATTATGGT | | | | | |
| OVK179 | CGACTCGAGACTCATTTGATTCACCTACAGTCTCTTTC | | | | | |
| OVK184 | ATAGGCGCGCCTTATCCTCGCTTATCATAAAACATTTTAAAATTTCC | | | | | |
| OVK221 | ACTGGATCCGCATTATATAGAGTATTATTTGAAAATATCTCAGTAAAAGA | | | | | |
| OVK222 | GATTGTTCCTCCTTGGTACCGAGG | | | | | |
| OVK223 | CCTCGGTACCAAGGAGGAACAATCATGAGGGGTGAGACGTTGAAAC | | | | | |
| OVK229 | CATGGATCCACTTTAAAAGCATTTGATGAACAATATCGT | | | | | |
| OVK230 | CATCTCGAGTTGAATACCTAAGCCTCGTAATTCTTTAAC | | | | | |
| OVK231 | ATCGTTGAAGTCTGCCCAATACTTCCTTTCGAAGGATATA | | | | | |
| OVK232 | GGAAGTATTGGGCAGACTTCAACGATTAATTTTTTGTTTGACTTTTAGTT | | | | | |
| OVK290 | CATGGATCCGAACGAGAAGTACATAACTCATCACAGCTAT | | | | | |
| OVK291 | TAGCTCGAGGGTTCTTTGATATGATTACAATTCCTAAACTTACATGTTTC | | | | | |
| OVK292 | CTAGGATCCATCTGAACCATGAACGAATCGAATTTAGAC | | | | | |
| OVK293 | ACTCTCGAGCTTATGCATGAGCTTTAGCTCAGGTG | | | | | |
| OVK471 | ACTGGATCCGATAACCAAGATAGATAAACCATGTT | | | | | |
| OVK472 | ACTGGCGCGCCTCATCGCATCCTTATAACAAATTGA | | | | | |
| OVK474 | ACTGGCGCGCCTATCACTAATATATATTTGTATTTTCTAAAGTATACTGTTCGATACGC | | | | | |
| OVK475 | CTAGGATCCTTCCTTATTTTAATTTGTTTTAGTTGAATGACAG | | | | | |
| OVK476 | ACTCTCGAGTCATTTGTGCCATGGCTTACTTCG | | | | | |
| OVK477 | ACTGGATCCTAGCTGCGGGCACGAAATCA | | | | | |
| OVK478 | ACTCTCGAGGTGCTGGTGGTTCTACAGAC | | | | | |
| OVK481 | ACTGGATCCGGATTAGAGCAATCGGCATATATGGATG | | | | | |
| OVK482 | ACTCTCGAGTCCAGCATGTAGAGCTTGACCACC | | | | | |
| OVK542 | ATCGGATCCGAATTTTTAATCAGTCCAAATAAAGGGGAACC | | | | | |
| OVK544 | ACTGGATCCAGATGGTAGGAAACAACTAATACAGTTCC | | | | | |
| OVK545 | ACTGGCGCGCCTTACATTGTATGGATTGGGTATCCGATAGCT | | | | | |
| OVK581 | ACTGGATCCGTGGTATGAAACTATCAGCAGCTTG | | | | | |
| OVK583 | CGGTGATATTCTCATTTTAGCCATTCAATTCACCATACCTTTCCCATAA | | | | | |
| OVK588 | AAACAATTCATCCAGTAAAATATAATATTT | | | | | |
| OVK591 | CATGGATCCGCAGCGCCATCTTCAAATGGTC | | | | | |
| OVK592 | TCAATTCCGGTGATATTCTCATTTTAGCCATCTAAATTGAATCACTCTCAATCATAC | | | | | |
| OVK594 | GTACTCGAGGCAATGGTCCATGTTCGATTT | | | | | |
| OVK600 | TAAGGGAGTAGTCTAAGAGAAAGATGTGAGAA | | | | | |
| OVK617 | CATGGATCCCTATTGGTATAGTGGCCTGA | | | | | |
| OVK618 | ACTCTCGAGACACTTTCATCTAATTCACCAC | | | | | |
| OVK621 | ACTGGATCCTGGTACAGGACGTTCAGACTATCC | | | | | |
| OVK622 | ACTCTCGAGAATCTAACTGTGCAACAAACGCTG | | | | | |
| OVK623 | ACTGGATCCGAAGATACTAAAGGCAAAGTGACGC | | | | | |
| OVK624 | ACTCTCGAGCATTCATTACGACTTTAGCAGGTGT | | | | | |
| **For Northern Blot** | | | | | | |
| **name** | **Sequence 5’-3’** | | | | **Targeted gene** | |
| OVK364 | GGTTGCTTAATTAGCGCAACACATAACGAATTTGCCTGAG | | | | *fapR* | |
| OVK540 | GCTGCTTGAGGGTGATTTATAATCCCCATTGATGATACTG | | | | *pdhD* | |
| **For RT-qPCR^(4)^** | | | | | | |
| **name** | **Sequence 5’-3’** | | | | **Targeted gene** | |
| OVK356 | CGGTCCAGACTCCTACGGGAGGCAGCA | | | | 16S rRNA | |
| OVK357 | GCGTGGACTACCAGGGTATCTAATCC | | | |  |  |
| OVK304 | CCCTTCATCACAGACCATGA | | | | *fapR* | |
| OVK305 | GATTGCGCTTTAACATTTGGA | | | |  |  |
| OVK469 | GCGGTGTTTTCCGTGTTACT | | | | *pdhB* | |
| OVK470 | TCCAGCAATCGCATCAAATA | | | |  |  |
| OVK306 | TGAACCATGAACGAATCGAA | | | | *plsX* | |
| OVK307 | CCTGCTGACACACATCCATC | | | |  |  |
| OVK324 | TGTAAAAGCAATCGCTGACG | | | | *fabH* | |
| OVK325 | AAATCCAGAACATGCTGCAA | | | |  |  |
| OVK400 | TGACATCAACGCCAAACATT | | | | *bfmBAA* | |
| OVK401 | GTTCTCGGTGGAGGTACTGC | | | |  |  |

(1) : FsX describe frameshift mutations. For example *lspA^R51H-FsX11^* indicated that R51 is the first amino acid modified, changed in Histidine. Fs=frameshift, X=stop codon. The number 11 following FsX indicate that 11 amino acids remain in the new open reading frame after the R51.

(2): Suppressors strains where selected by plating 50 μl of 82 independent over-night cultures of the *ΔcshA* strain on MH plate and incubated at 25°C. Fast appearing colonies (1 per culture) were subcloned once at 25°C and once at 37°C, before over-night cultures at 37°C for frozen stock. Genomic DNA where extracted for all strains and pooled by in 9 groups before library preparation (the 9 pool number are indicated and referred to the sample deposit on the GEO data base). Mutation where then reassigned to each specific strain by sanger sequencing. mutations in grey are secondary mutation not believed to be suppressors mutations as they are cumulated with mutated gene found several times in the screen or tested as in C53 and C31. Mutation in sup13 has not been tested.

In few cases, annotated as non-detected in the table, we were not able to defined the mutations. In the C31 strain, although a mutation have been detected, the reconstruction of the mutant did not show to be able to compensate the *ΔcshA* cold growth, another non-detected mutation is possibly present in this strain.

(3): allelic replacement was performed as previously described using a pyrEF/5-FOA counter selection system [[1](#_ENREF_1)]. The mutated allele with its surrounding sequence (to allow homologous recombination) where cloned on a pRLYC8 vector which bears a thermosensitive origin of replication and the pyrEF genes from *B. subtilis*. Once introduced in the desired strain (*ΔpyrEF*), the transformants are selected at 30°C and are subcloned at 42°C twice to select integration events. Excision events are then selected by subcloning integration events on MH medium supplemented with 5-FOA.

(4): oligonucleotides have been designed using eprimer3.

**References**

1. Redder P, Linder P (2012) New range of vectors with a stringent 5-fluoroorotic acid-based counterselection system for generating mutants by allelic replacement in Staphylococcus aureus. Appl Environ Microbiol 78: 3846-3854.

2. Giraud C, Hausmann S, Lemeille S, Prados J, Redder P, et al. (2015) The C-terminal region of the RNA helicase CshA is required for the interaction with the degradosome and turnover of bulk RNA in the opportunistic pathogen Staphylococcus aureus. RNA Biol 12: 658-674.

3. Khemici V, Prados J, Linder P, Redder P (2015) Decay-Initiating Endoribonucleolytic Cleavage by RNase Y Is Kept under Tight Control via Sequence Preference and Sub-cellular Localisation. PLoS Genet 11: e1005577.

4. Fey PD, Endres JL, Yajjala VK, Widhelm TJ, Boissy RJ, et al. (2013) A genetic resource for rapid and comprehensive phenotype screening of nonessential Staphylococcus aureus genes. MBio 4: e00537-00512.

5. Dyke KG, Jevons MP, Parker MT (1966) Penicillinase production and intrinsic resistance to penicillins in Staphylococcus aures. Lancet 1: 835-838.

6. Duthie ES, Lorenz LL (1952) Staphylococcal coagulase; mode of action and antigenicity. J Gen Microbiol 6: 95-107.

7. Tu Quoc PH, Genevaux P, Pajunen M, Savilahti H, Georgopoulos C, et al. (2007) Isolation and characterization of biofilm formation-defective mutants of Staphylococcus aureus. Infect Immun 75: 1079-1088.

8. Baek KT, Frees D, Renzoni A, Barras C, Rodriguez N, et al. (2013) Genetic variation in the Staphylococcus aureus 8325 strain lineage revealed by whole-genome sequencing. PLoS One 8: e77122.

9. Charpentier E, Anton AI, Barry P, Alfonso B, Fang Y, et al. (2004) Novel cassette-based shuttle vector system for gram-positive bacteria. Appl Environ Microbiol 70: 6076-6085.
