# Supplementary material for: The DEAD-box RNA helicase CshA is required for fatty acid homeostasis in Staphylococcus aureus
Source: PLoS Genet. 2020 Jul 30;16(7):e1008779. doi: 10.1371/journal.pgen.1008779 (PMC7392221; doi:10.1371/journal.pgen.1008779)
Supplement: Attachment — Submitted filename: Response to reviewers.docx [file pgen.1008779.s008.docx]

Reviewer's Responses to Questions

We are grateful to the reviewers for their careful analysis and positive judgement.

**Comments to the Authors:
Please note here if the review is uploaded as an attachment.**

Reviewer #1: Mutants in the DEAD-box RNA helicase CshA demonstrate a cold-sensitive phenotype. The paper represents a heavily genetic approach (appropriate for PLOS Genetics I would think) to understanding the underlying mechanism of this phenomenon. Supressor mutants were predominantly involved in the fatty acid biosynthesis pathway. The cshA mutant is defective in decreasing straight-chain fatty acids/increasing branched-chain fatty acids at low temperatures compared to the wild type. The suppressor mutants restore the wild type ratio of SCFAs to BCFAs at low temperatures. Inefficient degradation of pyruvate dehydrogenase mRNA in the mutant is proposed to play a major role in the phenomenon by favoring acetylCoA production that leads to unbalanced SCFA production. I find the work to be thorough and well written. It is not my policy to nit pick papers that I review. You do not need to convince this reviewer that staphylococcal membrane fatty acid homeostasis is an important and interesting topic.

Comments:

1. The switch in percentages between SCFA and BCFA is modest albeit reproducible apparently. Could the authors "drill down" a bit into their fatty acid analyses. For example could they look at individual fatty acid species and see how they are changing in the wild type, mutant, and suppressor mutants. They could calculate the average SCFA and BCFA carbon chain lengths to see if they shorten with lower temperature. Also is there branching switching to more anteiso (anteiso C15:0 most likely) at lower temperatures.

In the condition of our study, the decrease of SCFA in the wt strain from 37°C to 25°C, is associated with an increase in iso-odd. In contrast in *∆cshA* strain SCFA stay constant and iso-odd increased further at the expense of the two other types of BCFA. For suppressors the increase of BCFA could concern all or specific BCFA species depending of the mutation. According to the literature, chain length decreased with the temperature in the wt. *∆cshA* at 25°C clearly has a higher level of long chain fatty acids and in most of the suppressors, but not all, it tends to decrease. Because of the complexity of the observed effect we decided to simplify the data set and show only the BCFA/SCFA ratio in the main text, which corresponds to the major observed effect. We changed the text to also mention the effect of the chain length. We also have edited the S2_File. This excel file contains all the fatty acid analysis data sets. We added two sheets, one analyzing the fatty acid types and the other the chain length. In addition, in the comment for the S2_files, we added a table describing the major effect observed for each strain in each condition, proposing therefore a deeper analysis of the fatty acid profiles.

We added in the text

line 261: associated with a decrease of chain length,

line 274: and chain length decreases (S2_File).

line 276: while chain lengths are overall longer than in the wt strain (S2_File).

We added in the legend of figure4 (line 986): See S2_File for complete data set and analysis by fatty acid types and chain length.

Reviewer #2: The manuscript “The DEAD-box RNA helicase CshA is required for fatty acid homeostasis in Staphylococcus aureus” by Khemici et al presents a comprehensive analysis of suppressors of a cshA mutant, leading to the discovery that CshA modulates expression of fatty acid synthesis (FASII) genes and dictates membrane properties. Cold-resistant suppressors of a cshA mutant mapped mainly to fatty-acid related mutations. Using different approaches, authors identify Pdh activity as the likely primary cause of cshA cold-sensitivity. Pdh synthesizes acetyl-CoA, a FASII precursor; when in excess, acetyl-CoA primes the synthesis of more rigid straight-chain fatty acids (it outcompetes branched chain acyl-CoA primers), explaining the cold-sensitive phenotype. The numerous cshA suppressor mutations, which affect steps in fatty acid metabolism, then make sense. Authors further show that acetate supplementation, which increases acetyl-CoA production, also leads to cold-sensitivity in a wild type background. Cold sensitivity due to acetate addition is alleviated in the cshA suppressor backgrounds. Several suppressor mutations were affected in membrane composition but were not clearly linked to FASII (e.g., fakA, ltaS, lspA); the present results suggest the existence of novel roles of the affected genes. Altogether, the manuscript shows that CshA activity contributes to regulating membrane rigidity by controlling Pdh-catalyzed synthesis of acetyl-CoA, the precursor of straight-chain fatty acids.

This study provides important findings in understanding S. aureus membrane physiology and its regulation. The numerous investigative approaches, rigor, and breadth of the study convincingly support authors’ main hypotheses and expand the present understanding of fatty acid regulation and membrane homeostasis. The “honest” presentation is a pleasure to read. Authors explain that the observed phenotypes are seen on solid, but not liquid medium, and are medium-dependent. This seeming limitation is actually a pointer for explaining metabolic regulation.

Please note the following suggestions/questions:
1- The main “drawback” of this in-depth study is in presenting so much data! Perhaps authors could make certain sections more concise, e.g., shortening the sections on acetate-induced cold sensitivity (starting l 386) would simplify reading. Interpretations of phenotypes might be more appropriate for Discussion.

We have tried to make the manuscript as concise as possible. We agree that the manuscript is long due to the numerous mutants we obtained in the screen, and the different pathways at work in this study that need to be discussed. We believe that shortening will not help for the comprehension. Moving the phenotype interpretation into the discussion will increase the length of it which we would prefer to not do as you mention in the second note, it is already long enough.

2- The manuscript would benefit from including a model. Fig. 1 presents some results, but does not include subsequently presented information. It would be useful for readers to include the various pathways that compete for acetyl-CoA (the acetate connection as here, and also TCA, pigments and cell wall). The main concept (membrane fluidity as controlled by CshA) is exciting and could be depicted schematically. A shorter Discussion (there is redundancy with Results) with a summary Figure could better bring out the importance of the findings.

Fig1 has been modified accordingly by adding the connection with TCA, pigment and cell wall.

3- Does the ltaS mutation impact fatty acid composition as shown for other mutants?

We have not done fatty acid analysis for all mutations found in the screen, but decided to focus on some mutants that could be related to FAS II (all of them are presented in the manuscript). Unfortunately, *ltaS* mutations have not been studied.

4- L 122: Authors propose that “accumulation of the pdh mRNA in the DcshA strain at low temperature exacerbates the production of SCFA, at the expense of BCFA production”. Authors could discuss why their previous transcriptome study of a cshA mutant (Ref 9) did not detect changes in pdh.

The ref 9 presented a study of mRNA turnover in mutants of *cshA* at 37°C. We did observe changes in the *∆cshA*, as we mention in the text (line 330:” Interestingly we noticed previously that the *pdh* mRNA decayed slightly slower in a *∆cshA* strain at 37°C [9]”). These changes are not reported in the table 1 of the ref 9 because only mRNA stabilized above 2 fold are referred in this table. The *pdh* mRNA is, as noted, only slightly stabilized under the 2-fold threshold, and therefore the results concerning the *pdh* mRNA are found in the supplemental information of ref 9. The effect at 25°C observed here is stronger, which probably reflects that lower temperature strengthen RNA structures, thus increasing the requirement for an RNA helicase.

We added in the text (line 333): was further stabilized, above 2-fold, possibly because the RNA structure(s) that require CshA is strengthened at lower temperature (Fig 6C).

5- L 268 Could the fact that solid, but not liquid medium, selects for suppressors indicate that the TCA cycle, which also uses acetyl-CoA, may be less active in colonies? Have authors compared cold-sensitivity of cshA in anaerobic vs aerobic conditions?

The difference between solid versus liquid or the media dependency, is a very interesting question that probably reflect differences in central metabolism, with a key role of acetyl-CoA flow. Therefore, the activity of the TCA cycle is possibly at the center of these differences. However, we have not tried in this study to understand these observations or work on anaerobic condition. To go further it would be interesting to perform transcriptomic, metabolic, and fatty acid studies in the different conditions, which, we believe, is well above the scope of this study.

6- Authors state L 352 “lowering the FASII activity [with triclosan] compensates the over-expression of pdh”. How might this be explained?

We indeed showed that triclosan and FabD mutant, which both reduce the activity of one enzyme of the FASII (FabI and FabD, respectively), compensate the over-expression of the *pdh* mRNA. Fatty acid analysis was done for the *fabD* mutant (which shows increase of ante-iso and decrease of SCFA production (cf S2_file)) but not with triclosan. We therefore do not wish to speculate in the main text on the effect of triclosan. It could be direct due to a decrease activity of FabI, possibly decreasing chain length or an indirect effect of triclosan.

7- L510 For proper membrane function: “in Gram-positive bacteria, increased production of branched-chain fatty acid and decreased chain-length are the major responses”. Note that some Gram-positive like B subtilis express a phospholipid desaturase, which may allow rapid membrane adjustments (DOI: 10.1128/JB.185.10.3228-3231).

We added in the text (line 512): In some Gram-positive bacteria, such as *Bacillus subtilis* but not in *Staphylococcus aureus*, desaturase exists, and is proposed to be involved in short term adaptation [42,43] whereas the major response for long term adaptation relies on increased production of branched-chain fatty acids and decreased chain-length [15].

8- Table S1: C40: ltaSF64L-FsX2, what is FsX2?; Line 235 should read ahrC., line 261 “higly”. Check for numerous typos.

Thank you very much, typos have been checked.

ltaSF64L-FsX2: describes a frameshift mutation, using the ltaSF64L-FsX2 example: the first Amino acid affected is the F64 changed to leucine- Fs mean Frameshift and X stop codon. The number after the X refer to the remaining length of the new open reading frame. We added in the table S1 the description of the frameshift nomenclature.

Reviewer #3: The authors have an extensive history of influential manuscripts describing the roles performed by DEAD-box RNA helicases in the opportunistic pathogen Staphylococcus aureus. In the current manuscript the authors expand these studies by performing a genetic screen to identify suppressors of the cold growth defects exhibited by the delta-cshA mutant. Genome sequencing indicated that genes associated with fatty acid metabolism were altered in many of the isolated suprepressors. Extensive experimental analysis correlated the low temperature phenotype with CshA alteration of the membrane fatty acid composition at low temperature. These observations provide unique insights into bacterial perception and genetic/physiological response to low temperature stress associated with RNA helicase-mediated RNA decay and alteration of membrane composition.

Major comments

Mutation in the 82 suppressor strains suppresses the cshA cold sensitive phenotype allowing growth at low temperature. Many of these suppressors are in genes associated with fatty acid synthesis. Thus in these mutants a decrease in cellular ability to synthesize fatty acids, specifically BCFA, which be expected, synthesis of which would be required to make the membrane more fluid at low temperature. Thus should the cells not die at low temperature?

Indeed the different mutations obtained in the screen impact the fatty acids composition, but these specific mutations favor the BCFA production, as shown by our extensive fatty acid analysis, which we believe explains how they suppress the *cshA* mutant phenotype. This is our major conclusion from the results obtained in the genetic screen. Therefore, we are not sure to understand correctly this comment and especially the sentence: “in these mutants a decrease in cellular ability to synthesize fatty acids, specifically BCFA…”; as mentioned BCFA production does not decrease but in contrast is favored by the mutations.

Concerning death, we do not know the minimum level of BCFA required for survival in our condition. The *ΔcshA* strain has the lowest level of BCFA measured in this study, still they reach about 80%, which we proposed has an impact on growth but is sufficient for viability. We have not done a long-term experiment to measure a decrease in cell viability over time at low temperature (and unfortunately in the present time we can not perform this experiment since the laboratory is closed due covid-19).

While reading the manuscript I frequently wondered if, overall, the gene/membrane composition observations could result from a secondary effect from a more global regulator/pathway whose effect is enhanced at 25oC? The authors also struggle with this possibility at a number of points in the text.

It is true that we cannot totally rule out another effect. We were careful all along the text to not overinterpret the results. We proposed that disturbance of the acetyl-CoA flow, and especially overflow towards SCFA synthesis, explains in part the *ΔcshA* growth delay at low temperature. Nevertheless, the measurement of fatty acid composition and the many different genetic experiments, along with the effects observed by acetate addition in the media, which are compensated by the same mutations that compensate the 25°C growth of *ΔcshA*, are strong arguments for our interpretations.

For example, 13 of the hits occur upstream of the formation of malonyl-CoA. Thus, overall could the results not be explained by a defect in malonyl CoA utilization since it is used in numerous other pathways.

To our knowledge malonyl-CoA is used only for fatty acid synthesis. Should we read “acetyl-CoA”, which is indeed used in numerous other processes? In this latter case, 5 mutants (affecting the PDH complex, the proposed target) are upstream of acetyl-CoA production, and we agree that acetyl-CoA use, and in particular flow, is disturbed: this is our main hypothesis.

Line 188; The authors should check if the assumption that malonyl-CoA levels are reduced in the mutant. A 2-fold reduction in transcript level may not generate a decrease in product levels that is significant enough to alter carbon flow. In the manuscript, results that change transcript levels by 2-fold are frequently observed.

This is indeed an indirect read-out. But the change of transcription levels by itself (and not the possible subsequent change in product level) is the indication that malonyl-CoA levels vary in these mutant, since FapR activity, regulating the measured target genes, is under control of malonyl-CoA level. In the *acc* mutants, decreased *fapR* operon transcript levels indicate that its transcription is further repressed by FapR, indicative of a decrease of malonyl-CoA level (its repressor); In the *fabD* mutant transcription is higher, an indication that malonyl-CoA is accumulated, alleviating the repressor effect of FapR.

Potentially associated with the above comments, I wonder why the link between suppressor mutation and fatty acid/phospholipid composition was only analyzed for a subset of the mutants in the figures. I understand the reasoning to focus on the mutants in the initial parts of the pathway however a substantial proportion of the hits were in fakA (35) and ltas (12).

Indeed, not all mutants have been tested for fatty acid analysis, which would have been a very heavy and costly analysis. We had to select some of them and focus on the mutants in the initial parts of the pathway, as you mention. We have not selected the *ltaS* mutant which is highly interesting but may be difficult to explain and rely on an indirect effect. However, the *fakA* suppressor strain has been tested (line 474, fig4B and S4-fig).

With respect to fakA and ltas, how does their high prevalence in the data set fit into the overall conclusion that cshA indirectly affects membrane composition via pdh half-life since they act much below pdh.
Similar to the malonyl-CoA comment, could at least a portion of the results originate from fakA alteration of free fatty acid turnover? Although exogenous FAs do not shut off endogenous synthesis in Staphylococcus, could the results from the addition of exogenous fatty acids be insightful? In summary, further investigation of fakA and ltas could be insightful and further support the hypothesis.

We fully agree that additional effects, aside the *pdh* mRNA accumulation, could also participate to the phenotype. An alteration of FakA activity could be one of those. Concerning the number of mutations obtained in *fakA*, it is indeed provocative, but it also could be due to a bias of genetic screens as we mention in the text (line 603): “The high occurrence of mutations (in 35 strains) in *fakA* probably reflects two biases of the genetic screen: the efficiency of suppression in genes whose mutations have no obvious fitness cost and multiple ways to inactivate a gene”. Nevertheless, studying *fakA* and *ltaS* mutants will be interesting, but above the already extensive analysis presented here.

Significant emphasis was placed on alteration of the effects caused by exogenous acetate. Were common effects associated with acetate addition the best way to prove the hypothesis? In addition, while the effect of acetate on growth of various Staph strains was extensively studied at 37 and 25oC including delta-cshA and delta-cshA-delta-crt and in the presence or absence of triclosan (Figs. 8 and S2), why was the suppression of cold sensitivity by the other suppressors identified in the screen not investigated?

We do have investigated extensively a lot of suppressors. Results are shown in Fig 9. It is to note that all *ΔcshA* suppressors tested also compensate the acetate addition which strongly supports the hypothesis that the suppressors found in the genetic screen help counteracting a disturbed acetyl-CoA flow in the *ΔcshA* mutant.

Indicate why MH media was used in these studies. Importantly, was suppression of cold sensitivity also observed in: 1) liquid, 2) minimal media, and 3) rich media as well?
Similarly, why was 25oC chosen as the cold stress temperature? Are reduced or enhanced effects observed at higher and lower temperatures?
The answer to the media question above is provided on lines 266-269, the suppressors did not suppress cold sensitivity in liquid media (which one?). While an interesting observation, it is also potentially disturbing re the overall hypothesis presented. It would be beneficial if the authors could investigate the causes of this observation further.

Working at lower temperature than 25°C, below room temperature, would require specific equipment that we do not possess. As *ΔcshA* cold defect is readily observable at 25°C, and suppressor selection is possible at this temperature, we choose this set-up. MH is the standard media used in the lab, and was used here to be consistent with all our previously published data. We agree that studying the effect in different media or temperature would be interesting, which, as we believe, will possibly show that the differences are due to different states of central metabolism. However, to go further and understand the overall effects it would require transcriptomic metabolomics and fatty acids studies in different media and different temperatures, which is well above the scope of the present study.

We added in the material and methods (line 645): in MH medium, our standard lab medium, on MH plate at 25°C, a temperature where the *∆cshA* mutant still grows but with a marked delay.

Additional comments

Lines 125-127: It is not clear how this work connects “central metabolism” with fatty acid homeostasis. We proposed that the activity of the PDH complex, an actor of the central metabolism as it produces acetyl-CoA, affects the balance between BCFA and SCFA. This is discussed in the paragraph starting line 559 of the discussion.

Figure 1. Include branch sites leading to the formation of BCFA and SCFA forms. Associated with the ACC step, add AccC (2) and AccD (3) to be consistent. Indicate where Lipoteichoic acid synthase (12) contributes to the depicted biosynthetic pathway(?).

Figure1 has been changed accordingly.

Figure 2. Need an internal control for relative quantification in 2B.

As mentioned in material and methods, the same amount of total RNA was loaded for each strain, methylene blue coloration of the membrane was used as loading control. This experiment was done 4 times and is highly reproducible.

## Material and methods was changed to (line 715): Northern blot. Equal amounts of total RNA in each lane were separated on 1.2% agarose/formaldehyde gel and MOPS buffer, transferred on HybondN+ membrane (Amersham) by capillarity blotting and crosslinked using UV stratalinker 2400 (Stratagene). Loading was controlled by methylene blue coloration of the membrane, enabling to position 16S and 23S rRNA migration used as size markers. DNA oligonucleotides radiolabeled using T4PNK kinase and (γ32P)-ATP, were hybridized to the membrane in ExpressHyb hybridization solution (Clontech, Mountain View, CA, USA) over-night at 37°C and washed. Quantification was done by detection of the signal using a Typhoon FLA7000 phosphoimager (General Electric). Oligonucleotides are indicated in S1_Table.

Fig. 2D. Why was growth of cshB severely reduced by triclosan at both temperatures while wild type grew relatively normally?

We used this control to show that the suppressor effect of triclosan was specific for the *cshA* mutant, and that another cold sensitive DEAD-box RNA mutants is not compensated by triclosan, and indeed as you mention it has a negative impact on this strain but at present we do not have an explanation for the effect on the *cshB* mutant, because the molecular function of CshB is unknown.

Figure 3. Lines 243-249. Does ahrCN31stop also suppress the cold sensitivity through a dominant-negative effect in wild type?
How many of the identified suppressor genes convey suppression of the cold sensitivity to delta-cshA similar to ahrC and BCKD shown in this figure?

We have not tested the truncation, but only the full-length mutant (*ahrC^R44C^*). The *ahrC^N31stop^* mutant probably does not suppress the wild type strain as it is too short to bind DNA or to oligomerize (oligomerization site of AhrC lies in its C-terminal domain). We have not tested the effect of overexpression of all suppressor mutations. Note that fig2C (in *ΔcshA*) and fig9B (in *wt*) shows that overexpression of FapR, also leads to suppression.

Figure 4 and S4. Are the SCFA values statistically significant, both within each strain and between strains? Have changes of a similar magnitude been observed in FA ratios in Staph (e.g. reference 26) or other Gram-positive bacteria during temperature shift?
Also, is an increase in BCFA of ~4% (wt 37 vs 25oC) or a decrease of ~7% (delta-cshA 37 vs 25oC) sufficient to generate a lipid membrane that is functionally more or less fluid, respectively?

S2_file contains all the numerical data, average and standard deviation for all strains showing the reproducibility of the experiment. Our measurements of fatty acid at 37°C in MH, are similar to those observed in Ref 26 done in MH at 37°C, however they did not study effect of the temperature in this reference. The extent of changes depends on different parameters: media, temperature and strains. The specific conditions used in our study have not been tested in other studies, so it is difficult to compare them.

Figure 5. Clarify why FapR regulation of target gene expression was observed to not be involved in suppression of low temperature sensitivity, as indicated by the authors. As I understand the system, fapR mutation should have suppressed the low temperature sensitivity, however it did not and thus apparently invalidates the hypothesis. I also do not see the utility or corresponding insight gained by introducing the accC/D and pdhA mutations into the delta-cshA-delta-fapR mutant. From the nature of the suppressor screen would it not be anticipated that the accC/D and pdhA mutations, in the absence of delta-frpR, would be expected to confer suppression of the cold sensitivity in a delta-cshA background, similar to the results shown for other suppressor genes in Figure 3? Are accC/D and pdhA even regulated by FrpR?
Am I missing something?

As mentioned in the text, FapR is the repressor of fatty acid and phospholipid synthesis genes. Its deletion leads to an overexpression of its target genes. In *B. subtilis* deletion of *fapR* leads to cold sensitivity. It is the first time that the effect on temperature of a *∆fapR* mutant is studied in *Staphylococcus aureus* and as shown in this manuscript it does not lead to cold sensitivity in our condition. We discuss this difference between the two organisms in the discussion but do not know why this difference exists. This results therefore tends to invalidate the idea that FapR targets overexpression could be the reason of the cold sensitivity of the *∆cshA*.

Concerning the *acc* mutant: 2 hypotheses could be made, either the reason of the appearance of these mutations are to decrease product levels (acetyl-ACP then malonyl-CoA) or to decrease the transcription of FapR target genes (control by malonyl-CoA level) or both. Cumulating *∆fapR* with the *acc* mutants has no effect and therefore is not consistent with the second hypothesis. It is true that this is probably not surprising when looking at the overall study but we think it is worth to mention it.

Why are fapR transcript levels elevated to a similar degree in delta-cshA and delta-cshA plus the 3 phdA mutants while other suppressors reduce fapR levels, as shown in Fig. 2? Is there a unique insight provided by the difference between the FapR effect on the two sets of mutants?

Fig2 suggested that RNase Y mutant directly affects level of *pdh* mRNA by destabilization of the mRNA. The other mutants, where *pdh* mRNA levels are not changing, show that the effect on *pdh* mRNA steady state is not a common characteristic of the suppressors but specific to the RNase Y mutant.

Lines 333-336; How is the pdh northern blot in Fig. 6D indicative of the enhanced accumulation of the entire pdh operon?

The detected signal in the blot corresponds to the size of the entire operon containing 4 ORFs.

Lines 348-352; It is interesting that growth is inhibited by p-PDH at 37oC and the authors predict it reflects deregulation of another pathway that utilizes acetyl CoA.
Is this not evidence that the results could be explained by a defect in malonyl CoA utilization as it is used in numerous other pathways.

We are not sure to understand this comment: it is acetyl-CoA that is used by numerous pathways.

Fig. 6F. Would it be useful to repeat this experiment at 37oC? Possibly more insightful, how are fapR levels affected in the delta-rny strain?

It is true that we have not done RNA extraction from plates cultivated at 37°C, but only studied the effect at the temperature of interest. However, we performed RNAseq measurement both in *∆cshA* and the suppressor strain at 37°C and observed this effect. We do not wish to add those data as they have not been done in the specific condition of this study. We are nevertheless confident that it would be the same at 37°C but believe that it would not add much to the data presented here.

Figure 7. A potentially useful addition to this or other figures would include results from the artificial alteration of membrane fluidity caused by exposure to staphyloxanthine or benzyl alcohol. Would pdh transcript levels change in response to these compounds?

This is an interesting idea since we have no idea how *pdh* is controlled; however, the fact that it could be controlled by alteration of membrane fluidity was not supported by the RNA analysis in mutants where *pdh* levels are not changing. Understanding transcriptional control of *pdh* mRNA is an interesting issue that could be a potential follow-up of this study.

Figure 8. Provide as Supporting Information?

Conservation of phenotypes in different lineages of *S. aureus* is an important and recurrent question. Therefore, we believe it is interesting for many readers and would like to leave this figure in the main text.

Figure S2. How is LTA still formed in the C22 mutant?

The C22 strain is mutated has follow: *ltaS^T99K/T142K/P156T^*-We assumed that the mutant is not totally inactive, and still produces LTA.

“gram-negative” should be changed to “Gram-negative” in the entire document

Thank you very much, changes have been made.


Materials and Methods

Lines 640-644: Could the authors elaborate on the criteria used to: 1) select the initial suppressor colonies and 2) further purify candidates once at 25°C and why they were also purified at 37°C.

We added (line 647): Candidates were further purified once at 25°C, to selected true positive candidates and once at 37°C, to evaluate their capacity to grow at higher temperature.

Line 707; Could a more suitable control gene other than 16S rRNA be used for RT-qPCR quantification? The quoted paper identifies 16S rRNA as suitable in stationary phase cultures.

We have tested different references in the lab, and concluded that this one works well under the conditions we used. We have also used northern blot to assess mRNA level, and the measurement by the two techniques were very consistent. Changing the reference is not an easy task, especially in mutant affected in mRNA decay, and would require to repeat too many mRNA quantifications.

Lines 709-721: Provide further details regarding the RNA loading control used for Northern analysis and how quantification of signal intensities was performed.

Material and methods was changed to (line 715): **Northern blot.** Equal amounts of total RNA in each lane were separated on 1.2% agarose/formaldehyde gel and MOPS buffer, transferred on HybondN+ membrane (Amersham) by capillarity blotting and crosslinked using UV stratalinker 2400 (Stratagene). Loading was controlled by methylene blue coloration of the membrane, enabling to position 16S and 23S rRNA migration used as size markers. DNA oligonucleotides radiolabeled using T4PNK kinase and (γ32P)-ATP, were hybridized to the membrane in ExpressHyb hybridization solution (Clontech, Mountain View, CA, USA) over-night at 37°C and washed. Quantification was done by detection of the signal using a Typhoon FLA7000 phosphoimager (General Electric). Oligonucleotides are indicated in S1_Table.
Lines 726-729: Provide further details re the loading controls, standardization, identification and relative abundance determination related to fatty acid composition identification.

A detailed protocol provided by DSMZ was added in S3­­–File.

We added the link line 733: See S3_File for the detailed protocol provided by DSMZ.

Lines 730-740: Provide further details regarding how LRT was extracted and how loading was controlled for western blot analysis detection.

We do not understand this comment, we believe the protocol is already fully detailed. We unfortunately do not possess an antibody that could be used as a loading control in this experiment.
